# Alexithymia moderates brain function during suppression of negative emotions in fibromyalgia

**DOI:** 10.1097/j.pain.0000000000004109

**Published:** 2026-09-01

**Authors:** Amaia Aguirre de Cárcer Vidal, Nell Norman-Nott, Sylvia M. Gustin, Yann Quidé

**Affiliations:** a NeuroRecovery Research Hub, The University of New South Wales (UNSW) Sydney, Sydney, NSW, Australia; b Centre for Pain IMPACT, Neuroscience Research Australia, Randwick, NSW, Australia; c School of Psychology and Counselling, Queensland University of Technology, Brisbane, QLD, Australia

**Keywords:** Fibromyalgia, Emotion regulation, Suppression, Alexithymia, Functional connectivity

## Abstract

Fibromyalgia is associated with altered brain function and emotion regulation difficulties. Alexithymia is common in fibromyalgia and may further affect emotion regulation in this population. This study examined the interplay of alexithymia and fibromyalgia on brain function during suppression of negative emotions. Twenty-seven female participants with fibromyalgia and 30 controls performed an emotion regulation task while undergoing functional magnetic resonance imaging. Alexithymia was assessed using the Toronto Alexithymia Scale (TAS). A series of whole-brain multiple linear regressions was performed to estimate the effects of group, alexithymia, and their interaction on activation and seed-based task-related functional connectivity. Reduced activation in the left middle frontal gyrus during negative emotion suppression, as well as reduced left amygdala–right temporo-parietal junction connectivity, increased left anterior insula–right posterior insula connectivity, and increased left anterior cingulate cortex–precuneus and inferior parietal lobule connectivity were evident in fibromyalgia. Increasing alexithymia was associated to stronger left anterior insula–inferior parietal lobule connectivity, across all groups. After significant association between the group-by-TAS interaction and right amygdala–lingual gyri connectivity, moderation analysis indicated that increasing alexithymia was associated with reduced connectivity in healthy controls but not in fibromyalgia. This study highlights disrupted integration between emotional, interoceptive, and self-referential networks as a key feature of negative emotion suppression in fibromyalgia. Alexithymia moderates these disruptions by reducing emotional clarity and increasing internal focus. Interventions enhancing interoceptive awareness, fostering emotional understanding, and strengthening cognitive regulation may help normalise neural function and improve emotional functioning, especially in individuals with elevated alexithymia.

## Abstract 2

Supplemental Digital Content is Available in the Text.

Fibromyalgia alters brain networks for negative emotion suppression; alexithymia further moderates suppression-related brain function and connectivity, highlighting impaired emotional processing and interoception as intervention targets.

## 1. Introduction

Fibromyalgia is a chronic, idiopathic condition characterised by widespread musculoskeletal pain without identifiable tissue damage or inflammation.^82^ Prevalence ranges from 0.2% to 6.6%,^43^ depending on diagnostic criteria,^26^ and disproportionately affects women. Beyond pain, fibromyalgia is frequently accompanied by depression, anxiety, and emotion regulation difficulties,^67^ alongside altered brain function during emotion processing and regulation.^6^ Emotion regulation refers to the monitoring, evaluation, and modification of emotional responses to achieve behavioural goals.^29^ One contributing factor in emotion regulation difficulties is alexithymia, a related but distinct construct characterised by difficulties identifying and describing emotional states and externally oriented thinking.^5,66^ Whereas emotion regulation involves managing emotional responses, alexithymia reflects impaired emotional awareness that may influence regulation processes.

Neuroimaging research links alexithymia to functional alterations in brain regions involved in detecting emotionally salient information, interoceptive processing of internal bodily signals, and regulatory control.^73^ Heightened amygdala, anterior insula, and anterior cingulate cortex (ACC) activity, alongside reduced medial and dorsomedial prefrontal activation during emotional appraisal and regulation.^73^ These findings indicate that alexithymia disrupts the mapping of interoceptive signals onto conscious emotional awareness. In fibromyalgia, alexithymia is consistently elevated, even when controlling for comorbid depression and anxiety,^31,63,68^ suggesting a role in altered interoception and affective salience processing. However, its role in influencing neural processes underlying emotion regulation in fibromyalgia remains unclear.

The Fibromyalgia Integrative Training for Self-regulation and Salience (FITSS) model^55^ provides a framework for these alterations, proposing that dysfunction within the salience network, comprising the ACC, anterior insula, and amygdala, leads to maladaptive emotional and somatic processing and emotion regulation difficulties.^71^ This network interacts with the central executive network, including the dorsolateral prefrontal cortex (dlPFC), supporting top-down control,^19,42^ and with the default mode network (DMN), encompassing the medial prefrontal cortex (mPFC), posterior cingulate cortex, bilateral temporo-parietal junctions and the hippocampus, involved in self-referential processing.^61^ Given its role in disrupted interoceptive-affective integration, alexithymia may contribute to variability in salience–prefrontal interactions during emotion regulation in people with fibromyalgia.

One experimental strategy to examine these mechanisms is emotion suppression. Suppression involves inhibition of emotional expression via top-down prefrontal modulation of limbic responses.^30^ Because suppression requires internal emotional state monitoring while regulating behavioural expression, it is cognitively demanding and challenging in individuals with altered interoceptive processing, that is, disrupted internal bodily signals processing, including those with fibromyalgia.^6,64^ Accordingly, alexithymia may shape the neural processes engaged during emotion suppression in fibromyalgia.^14,35,72^

The current study examined brain activation and functional connectivity during suppression of negative emotions in women with fibromyalgia compared with controls and tested alexithymia as a moderator of these neural responses. Fibromyalgia was hypothesised to be associated with heightened salience activation and connectivity (ACC, anterior insula, amygdala) during suppression, alongside stronger coupling with executive control (dlPFC) and default mode networks (mPFC) reflecting increased regulatory demand. Higher alexithymia was further expected to be associated with exaggerated limbic responsivity and reduced prefrontal engagement, indicating less efficient top-down modulation during emotion suppression.

## 2. Material and methods

### 2.1. Participants

Data from 33 female participants with fibromyalgia and 33 female participants without a chronic pain condition (controls) were obtained from the same publicly available OpenNeuro dataset, ds004144 (https://openneuro.org/datasets/ds004144/versions/1.0.2).^8^ This dataset was selected because it combines task-based fMRI of emotion regulation with validated measures of alexithymia and detailed clinical characterization within the same participants, enabling the present research question to be addressed. Recruitment and clinical assessment details are available elsewhere.^7^ Participants were adults (18-50 years old) with at least elementary education. Diagnoses of fibromyalgia were given by a rheumatologist or an internal medicine specialist, confirmed by meeting both the American College of Rheumatology 1990 (tender points)^80^ and 2016 criteria (widespread pain and symptom severity).^79^ Participants were recruited through educational talks about fibromyalgia at the National Institute of Psychiatry in Mexico City, the Mexican Foundation for Fibromyalgia, and through posters and social media advertisements. Exclusion criteria included the presence of a major psychiatric, cardiovascular, neurological, or other pain conditions.^7^ All participants answered a standardized instrument to measure socio-economic level in Mexican households, the Socio-economic levels Questionnaire of the Mexican Association of Market Research and Public Opinion Agencies Questionnaire (AMAI NSE 8 × 7). Details are presented Table 1. The protocol was approved by the Research Ethics Committee of the National Institute of Psychiatry “Ramón de la Fuente Muñiz” in Mexico City, in accordance with the Declaration of Helsinki, and all participants provided written informed consent.

**Table 1:** Socio-demographic characteristics of the sample.

	Controls (n = 30)		Fibromyalgia (N = 27)		Statistics		
	Mean	SD	Mean	SD	t-value/X^2^	df	*P*
Age, y	41.20	6.22	41.56	6.48	−0.211	55	0.833
Education, y	16.50	3.72	15.57	3.63	0.969	55	0.337
Marital status, S/M/SE/W	7/18/4/1		9/13/3/2		1379	3	0.711
Occupation, PE/BU/T/L/H/ST/P/UN/Other	16/3/2/1/5/1/2		9/3/2/2/9/2/0		5.627	6	0.466
Occupation pattern*, FT/HTF/HTI	15/5/5		9/1/10		5.344	2	0.069
Socioeconomic level†, D/D+/C−/C/C+/AB	1/1/5/0/13/10		0/6/6/1/5/9		9.138	5	0.104
Probable menstrual cycle at scanning time, FL/LU/PM/UNK	14/12/1/3		12/8/3/3		1.677	3	0.642
Handedness, L/A/R	0/1/29		0/1/26		0.006	1	1.000

* Among those who work, controls, N = 27, fibromyalgia, N = 23.

† The Socio-economic levels Questionnaire of the Mexican Association of Market Research and Public Opinion Agencies Questionnaire (AMAI NSE 8 × 7) indicates that A/B is the highest economic status category and E is the lowest.

df, degrees of freedom; FL/LU/PM/UNK, follicular/luteal/postmenopausal/unknown (hysterectomy); FT/HTF/HTI, full time/half time fixed schedule/half time irregular; L/A/R, left handed/ambidextrous/right handed; PE/BU/T/L/H/ST/P/UN/Other, professional or executive/business/technician/labourer/housewife/student/pensioner/unemployed/other; S/M/SE/W, single/married or de facto/separated or divorced/widowed; SD, standard deviation.

### 2.2. Clinical scales

No longer than 2 weeks before the imaging session, participants completed a comprehensive clinical and psychological assessment. The current health status and symptom severity associated with fibromyalgia were assessed using the 20-item Spanish version of the Fibromyalgia Impact Questionnaire.^75^ Pain from a dimensional perspective was evaluated using the 78-item Spanish version of the McGill pain questionnaire.^44^ Duration and current treatment for fibromyalgia were recorded using the Fibromyalgia General Questionnaire.

### 2.3. Other clinical scales

Levels of alexithymia were measured using the 20 items self-rated Toronto Alexithymia Scale (TAS).^5^ Although subdomains of alexithymia can be derived from the TAS (difficulty identifying emotions, difficulty expressing emotions, and externally oriented thought), only the total TAS score was used for focal analyses.

Severity of depressive symptoms were evaluated using the Spanish version of the Hamilton Depression Rating Scale.^15^ The version used comprised 21 items, each item being scored on a Likert-type scale (scores ranging 0-4). The Spanish version of the Hamilton Anxiety Rating Scale^13^ was used to measure the severity of anxiety symptoms experienced. This 14-item Likert-type scale scored the intensity, frequency, and dysfunction caused by symptoms (scores ranging 0-4).

### 2.4. Functional magnetic resonance imaging task

Participants performed a validated emotion processing and regulation task.^6,74^ The task required the participants to respond to 3 different instructions (attend, reappraise, suppress) and for 3 emotionally valenced stimuli (neutral, positive, negative pictures from the International Affective Picture System)^38^ for a total of 7 conditions (attend neutral, attend negative, attend positive, reappraise negative, reappraise positive, suppress negative, suppress positive). Images were selected in the original dataset based on normative valence and arousal ratings to represent positive, negative, and neutral categories.^7^ The specific image content was determined by the original dataset and was not independently manipulated in the present study. The stimuli consisted predominantly of human scenes depicting pleasant, unpleasant, or neutral emotional content, with picture pairs matched on valence, arousal, activity, and scene complexity.^6,39^ In addition, stimulus properties (eg, luminance, content) were not independently controlled in the present study, as images were drawn from a preexisting dataset. The task was implemented in a block design, with each block representing each condition, and each condition was repeated 3 times (3 blocks/condition). Each block contained the instruction for that block (attend, increase, decrease, or suppress), followed by 4 pictures, and 3 evaluation screens with a visual analogue scale on each. Blocks were separated by a fixation cross of varying length (8,000-14,500 ms). Using visual analogue scales (ranging 0-10), participants rated their current subjective activation/arousal (“How awake do you feel at this moment?”). We interpret this measure as reflecting perceived arousal or activation, although it may also capture aspects of alertness and fatigue. Consequently, it should not be considered a direct or validated measure of emotional intensity. The affective state item (“How do you feel at this moment?”) did not include explicit valence anchors and may, therefore, reflect a broad subjective appraisal rather than a strictly standardised measure of positive or negative affect. Finally, participants were also asked about the physical pain at that moment (“How intense is the pain at this moment?”). Blocks were presented pseudorandomized (first a neutral block, then 6 emotional blocks in random order, then 7 blocks including both emotional and neutral blocks in random order, then 6 emotional blocks in random order, and finally a neutral block). This study focused solely on the suppression of negative emotion blocks.

### 2.5. Magnetic resonance imaging

#### 2.5.1. Images acquisition and processing

Whole-brain T1-weighted and T2*-weighted scans were acquired using a Philips 3T Ingenia scanner (Philips Healthcare, Best, The Netherlands) with a 32-channel phased array coil. As described elsewhere,^6,7^ scanning parameters for the three-dimensional T1-weighted FFE SENSE sequence were repetition time (TR) = 7.0 milliseconds, echo time (TE) = 3.5 milliseconds, flip angle = 8°, field of view (FOV) = 240 mm^2^, matrix = 240 × 240 mm, number of slices = 180, gap = 0, plane = sagittal, slice thickness = 1.0 mm, and voxel size = 1 × 1 × 1 mm. Scanning parameters for the emotion regulation task sequence were gradient-echo EPI, TR/TE = 2000/30.001 milliseconds, FA = 75°, matrix = 80 × 80, FOV = 240 mm^2^, voxel size = 3 × 3 × 3 mm, slice thickness = 3.0 mm, slice acquisition order = interleaved (ascending), number of slices = 36, phase encoding direction = AP, volumes = 834, and total task duration = 27.8 minutes.

Image processing followed the CONN toolbox's default pipeline (release 22.v2407; https://web.conn-toolbox.org)^78^ for SPM12 (v7771; Wellcome Trust Centre for Neuroimaging, London, United Kingdom; http://www.fil.ion.ucl.ac.uk/spm) in Matlab r2024a (Mathworks Inc., Sherborn, MA). Functional and anatomical data were preprocessed using a modular preprocessing pipeline including realignment with correction of susceptibility distortion interactions, slice timing correction, outlier detection, direct segmentation and Montreal Neurologic Institute (MNI)-space normalization, smoothing, and regression of temporal components. Functional data were realigned using SPM realign & unwarp procedure, where all scans were coregistered to a reference image (first scan of the first session) using a least squares approach and a 6 parameter (rigid body) transformation and resampled using b-spline interpolation to correct for motion and magnetic susceptibility interactions. Temporal misalignment between different slices of the functional data was corrected after SPM slice-timing correction procedure, using sinc temporal interpolation to resample each slice BOLD time series to a common mid-acquisition time. Potential outlier scans were identified using ART as acquisitions with framewise displacement above 2 mm or global BOLD signal changes above 9 standard deviations,^57^ and a reference BOLD image was computed for each subject by averaging all scans excluding outliers. Functional and anatomical data were normalized into standard MNI space, segmented into grey matter, white matter, and cerebrospinal fluid (CSF) tissue classes, and resampled to 2-mm isotropic voxels after a direct normalization procedure using SPM unified segmentation and normalization algorithm with the default IXI-549 tissue probability map template. Functional data were smoothed using spatial convolution with a Gaussian kernel of 8-mm full width half maximum. Last, components were removed from the BOLD signal time series using a separate linear regression model at each individual voxel.

In addition, functional data were denoised using a standard denoising pipeline including the regression of potential confounding effects characterized by white matter time series (5 CompCor noise components), CSF time series (5 CompCor noise components), motion parameters and their first order derivatives (12 factors), and linear trends (2 factors) within each functional run, followed by high-pass frequency filtering of the BOLD time series above 0.008 Hz. CompCor^9^ noise components within white matter and CSF were estimated by computing the average BOLD signal as well as the largest principal components orthogonal to the BOLD average, motion parameters within each subject's eroded segmentation masks.

After preprocessing, 5 participants with fibromyalgia and 3 controls were excluded because of excessive movements, and stimuli onset times were missing for 1 fibromyalgia participant. Mean framewise displacements did not statistically differ between the groups (*t*[*55*] = −1.488, *P* = 0.142, range [0.092,0.450]). The final sample consisted of 27 female participants with fibromyalgia and 30 controls.

#### 2.5.2. Seed regions

Partly consistent with^6^ we selected the left and right amygdalae, anterior insulae, and ACC, core nodes of the salience network, as seed regions for this study. The amygdala seed regions (left: 220 voxels; right: 248 voxels) were extracted from the *Automated Anatomical Labeling* atlas (*aal*)^70^ available with the Marsbar toolbox (v0.45).^11^ The anterior insula seed regions were extracted from the Hammers atlas,^23,32^ with the left anterior insula seed made of regions number 86 and 92 (792 voxels) and the right anterior insula seed made of the regions number 87 and 93 (809 voxels). Finally, as in^6^, 5-mm spheres around published coordinates (MNI [±6, 36, 10]; 81 voxels) were created for the ACC seed regions.

#### 2.5.3. Task-related functional connectivity

The generalized Psycho-Physiological Interactions toolbox (gPPI, v7.12, https://www.nitrc.org/projects/gppi)^45^ was used to derive the individual-level whole-brain task-related functional connectivity maps during the suppression of negative emotions for each of the 6 seed regions. These analyses were performed on preprocessed and denoised data generated using the CONN toolbox. The gPPI toolbox provides improved sensitivity and specificity compared with standard PPI implementations in SPM^27,28^ and allows flexible modelling of condition-specific task-dependent connectivity. For each subject, the mean BOLD time series was extracted from all voxels within each seed region and deconvolved from the canonical hemodynamic response function to estimate the underlying neuronal signal (physiological regressor). The psychological regressor corresponded specifically to the suppression of negative emotion condition and was constructed by convolving the relevant condition onset times with the canonical hemodynamic response function. The psychophysiological interaction term was computed as the product of the physiological and psychological regressors. Individual gPPI contrast images were then generated automatically by the toolbox for each seed region. These subject-level gPPI connectivity maps were subsequently entered into second-level whole-brain analyses, as described below. No additional nuisance regressors were included at the gPPI modelling stage, as motion- and physiology-related confounds had already been addressed during preprocessing and denoising in CONN.

### 2.6. Statistical analyses

Because of the relatively small sample size, second-level (group) analyses were performed using the Statistical non-Parametric Mapping toolbox (SnPM13.1.09; http://www.nisox.org/Software/SnPM13/) for SPM12.^50^ This toolbox uses permutation tests, avoiding normality assumptions and reducing false positives in small samples.^22,81^ Multiple linear regressions tested the main effects of group (controls vs fibromyalgia), levels of alexithymia (TAS total), and their interaction (the product of group × mean-centred TAS total score) on brain activation and functional connectivity during suppression of negative emotions (1 model per seed). SnPM13 default parameters were used to define the cluster-forming threshold, and family-wise error correction was applied to the cluster statistics (*pFWEc* < 0.05). Cluster-based parameters were set to perform 10,000 permutations, and variance smoothing was not applied (set to [0,0,0]). After whole-brain analyses, raw signal at the peak of significant clusters were extracted for further analyses using R (version 4.4.0)^60^ in RStudio (version 2024.04.1 + 748).^56^ Because of the relatively small sample size and the number of seed regions investigated, gPPI analyses were exploratory and no additional correction to account for the number of seed regions studied were applied.

In case of significant associations between the group-by-TAS interaction and brain activation or functional connectivity, moderation analyses were performed using the “interactions” R package (v1.1.5)^40^ on the extracted signal at the cluster peaks. Two sets of moderation analyses were performed. In the first, the effects of TAS total score (independent variable) on brain function or connectivity (dependent variable) were tested for each group (moderator). In the second, the effects of group (independent variable) were tested at 3 levels of TAS total scores (moderator): at 1 standard deviation (SD) below the average TAS total score (low TAS total score), at average TAS total score, and at 1 SD above the average TAS total score (high TAS total score).^18^ The Davidson–MacKinnon correction (HC3) was used to account for heteroskedasticity^33^ using the R package “sandwich” (v3.2.2).^84,85^ Statistical significance was set at a threshold of *P* < 0.05. A power analysis using G*Power 3.1.9.2^24^ indicated that a minimum of 54 participants was necessary for these moderation analyses (F3,50 = 2.79, λ = 18.90), confirming that our final sample (n = 57) was of sufficient size to achieve 95% power for detecting a large effect (*f*^*2*^ = 0.35) for 3 predictors (group, TAS total score, interaction) at α = 0.05.

## 3. Results

### 3.1. Sample characteristics

Sociodemographic and clinical details are presented in Tables 1 and 2, respectively. The final sample comprised 27 women with fibromyalgia (mean age = 41.56 years, SD = 6.48) and 30 pain-free controls (mean age = 41.20 years, SD = 6.22). The fibromyalgia and the control groups were statistically matched for age, handedness, years of education, occupation levels, socio-economical levels, and probable menstrual cycles (Table 1). Compared with the control group, the fibromyalgia group showed higher levels of alexithymia, depression, and anxiety symptoms (Table 2).

**Table 2:** Clinical characteristics of the sample.

	Controls (n = 30)		Fibromyalgia (N = 27)		Statistics		
	Mean	SD	Mean	SD	t-value/X^2^	df	*P*
Time since diagnosis (y)	—	—	4.10	4.75	—	—	—
Time since symptoms (y)	—	—	8.26	10.69	—	—	—
Current medication received—yes/no	—	—	24/3		—	—	—
Current medication received—number of medications used daily 0/1/2/3/4/5	—	—	8/13/5/1/0/0		—	—	—
Pain during interview (VAS)*	1.30	3.56	44.26	20.09	**−10.957**	**27.464**	**<0.001**
FIQ total score	—	—	32.01	9.58	—	—	—
McGill Pain questionnaire—sensory dimension	—	—	42.92	12.53	—	—	—
McGill Pain questionnaire—affective dimension	—	—	7.38	3.55	—	—	—
McGill Pain questionnaire—evaluative dimension	—	—	3.23	1.77	—	—	—
McGill Pain questionnaire—miscellaneous dimension	—	—	8.54	4.17	—	—	—
McGill Pain questionnaire—total score	—	—	43.23	12.50	—	—	—
Total widespread index (American College of Rheumatology criteria 2016)*	0.97	1.43	11.41	4.05	**−12.705**	**31.770**	**<0.001**
Total symptom severity score (American College of Rheumatology criteria 2016)*	1.47	1.48	8.19	2.59	**−11.860**	**40.412**	**<0.001**
TAS total score	39.40	12.18	55.33	20.30	**−3.545**	**41.663**	**<0.001**
HAMD total score	1.33	1.99	14.67	6.52	**−10.204**	**30.342**	**<0.001**
HAMA total score	2.33	2.50	20.63	7.02	**−12.837**	**31.887**	**<0.001**

* Five healthy controls reported some pain during the interview, because of high physical activity in the previous days. None of the participant from the control group experienced any chronic pain condition.

df, degrees of freedom; FIQ, fibromyalgia impact questionnaire; HAMA, Hamilton anxiety rating scale; HAMD, Hamilton depression rating scale; SD, standard deviation; TAS, Toronto alexithymia scale; VAS, visual analogue scale.

Significant group differences are in bold.

### 3.2. Behavioural results

Behavioural results are presented in Supplementary Material (Table S1 and Fig. S1, https://links.lww.com/PAIN/C570). The fibromyalgia group reported significantly lower levels of emotional valence ratings (ie, more negative affect) during conditions involving negative emotions (attend, reappraise, suppress) compared with controls and reported higher levels of arousal while attending negative emotions. The fibromyalgia group reported significantly higher levels of pain during all conditions compared with controls.

### 3.3. Brain activation

The linear regression model indicated a significant association between brain activation during suppression of negative emotions and group (*pFWEc* = 0.042), but not with TAS total score or the group-by-TAS interaction. During the suppression of negative emotions, female participants with fibromyalgia showed significantly weaker activation in the left middle frontal gyrus compared with controls, suggesting reduced regulatory control (Table 3A and Fig. 1).

**Table 3:** Peaks of clusters showing significant associations between group, Toronto alexithymia scale total scores, or their interaction on (A) brain activation, and (B) task-related functional connectivity with the selected seed regions during suppression of negative emotions.

	Seed	Effect	Hem	Cluster region	MNI coordinates			Cluster size (voxels)	Peak *t*-statistic	Cluster *P* (FWEc)
					x	y	z			
A. Brain activation										
	—	Group	L	Middle frontal gyrus; precentral gyrus	**−48**	**6**	**38**	**255**	**4.29**	**0.042**
					−42	14	40		4.29	
					−48	24	36		3.92	
		TAS	—	—	—	—	—	—	—	—
		Interaction	—	—	—	—	—	—	—	—
B. Task-related functional connectivity										
	L Amy	Group	R	Middle/superior temporal gyri; angular gyrus	**56**	**−50**	**22**	**223**	**4.73**	**0.042**
					44	−48	28		3.76	
		TAS	—	—	—	—	—	—	—	—
		Interaction	—	—	—	—	—	—	—	—
	R Amy	Group	—	—	—	—	—	—	—	—
		TAS	L/R	Lingual gyrus; cerebellar IV-V lobules	**16**	**−58**	**−8**	**319**	**4.72**	**0.028**
					−16	−54	−10		4.57	
					−16	−42	−10		3.89	
		Interaction	L/R	Lingual gyrus; cerebellar IV-V lobules	**−16**	**−54**	**−10**	**277**	**4.47**	**0.046**
					18	−58	−8		4.45	
					−16	−42	−10		4.09	
	L AIns	Group	R	Posterior insula; Rolandic operculum	**48**	**−4**	**12**	**366**	**4.53**	**0.011**
					36	−14	12		4.45	
					36	−32	18		3.47	
		TAS	L	Inferior parietal gyrus	**−50**	**−46**	**48**	**301**	**4.69**	**0.034**
					−40	−52	52		4.36	
		Interaction	—	—	—	—	—	—	—	—
	R AIns	Group	—	—	—	—	—	—	—	—
		TAS	—	—	—	—	—	—	—	—
		Interaction	—	—	—	—	—	—	—	—
	L ACC	Group	L/R	Precuneus	**−2**	**−70**	**38**	**388**	**4.72**	**0.012**
					2	−60	42		3.96	
					−2	−58	32		3.55	
			L	Inferior parietal gyrus	**−42**	**−50**	**34**	**387**	**4.35**	**0.012**
					−52	−58	24		3.90	
					−46	−64	40		3.87	
		TAS	—	—	—	—	—	—	—	—
		Interaction	—	—	—	—	—	—	—	—
	R ACC	Group	—	—	—	—	—	—	—	—
		TAS	—	—	—	—	—	—	—	—
		Interaction	—	—	—	—	—	—	—	—

Main peaks within the cluster of interest are in bold.

FWEc, family-wise error correction at the cluster level; Hem, hemisphere; Interac, Group-by-TAS interaction; L/R ACC, left/right anterior cingulate cortex; L/R AIns, left/right anterior insula; L/R Amy, left/right amygdala; L, left; MNI, Montreal Neurologic Institute; R, right; TAS, Toronto Alexithymia Scale total score.

**Figure 1.**
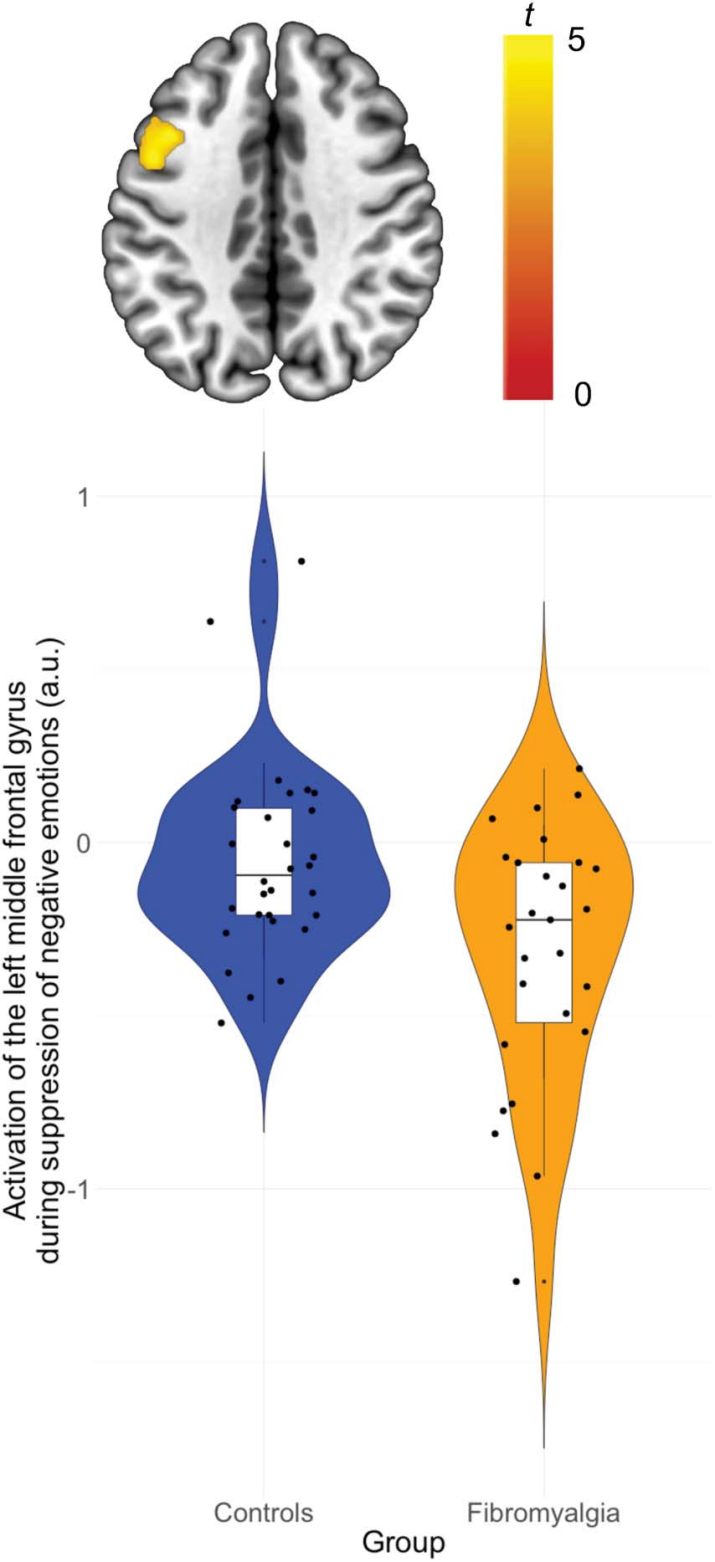
Group difference in brain activation during suppression of negative emotions. The group of female participants with fibromyalgia (in yellow) showed reduced activation in the left middle frontal gyrus compared with the group of pain-free controls (in blue). Statistical significance was set at a voxel-wise threshold of *P* = 0.001 uncorrected, to which a family-wise error (FWE) correction was applied on cluster statistics (*p*[*FWEc*] < 0.05). Left is the left of the figure; a.u., arbitrary units; colour-bar represents t-statistics.

### 3.4. Task-related functional connectivity

#### 3.4.1. Left amygdala seed

The whole-brain multiple linear regression model showed a significant association between group and connectivity between the left amygdala seed and the right temporo-parietal junction (TPJ; *pFWEc* = 0.042) during suppression of negative emotions (Table 3B and Fig. 2A). Female participants with fibromyalgia showed weaker left amygdala–right TPJ connectivity compared with controls, suggesting impaired integration. Connectivity with the left amygdala seed was not significantly associated with TAS total scores or the group-by-TAS interaction.

**Figure 2.**
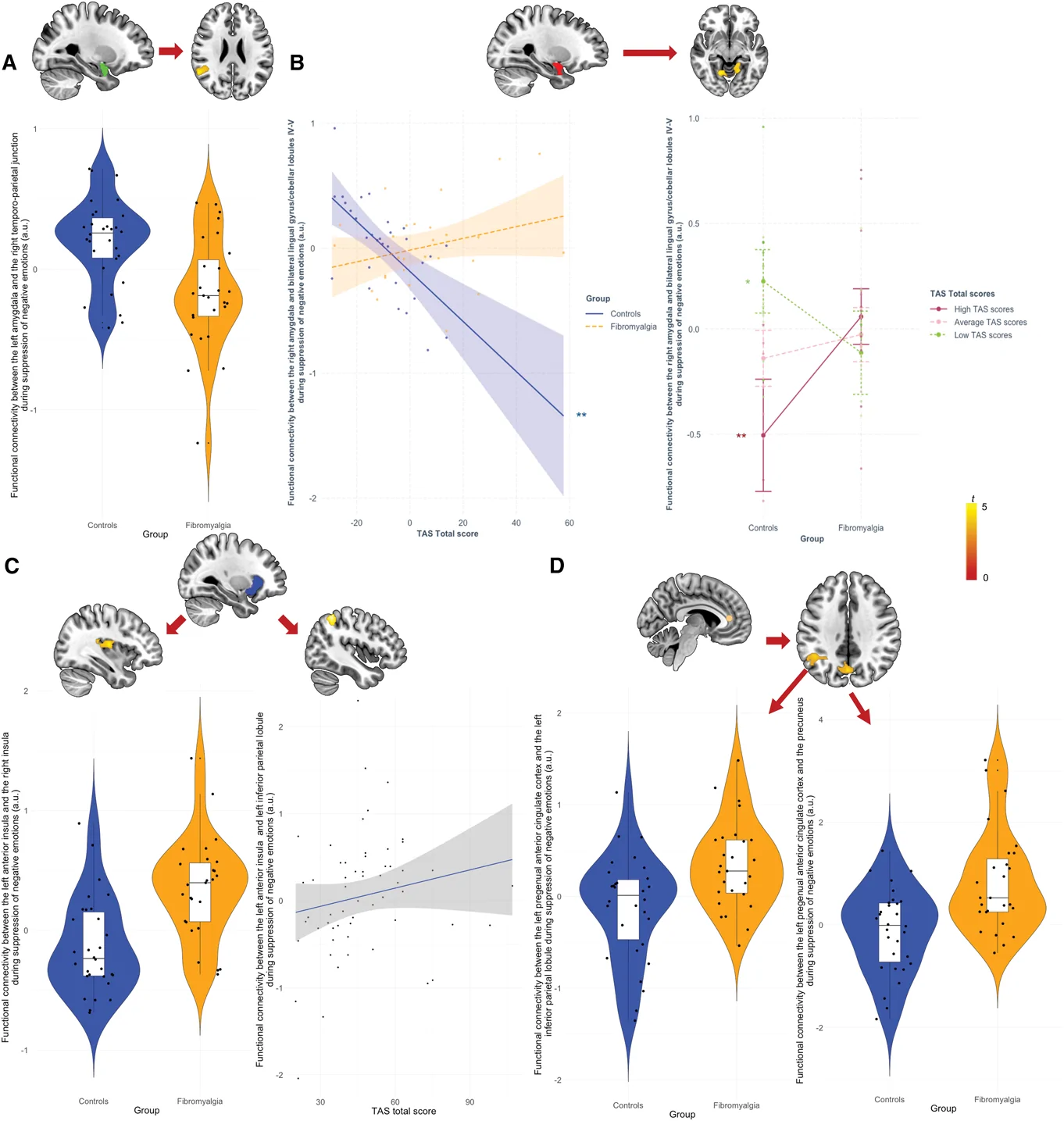
Group differences in task-related functional connectivity during suppression of negative emotions. (A) Compared with pain-free controls (in blue), the fibromyalgia group (in yellow) showed weaker connectivity between the left amygdala seed (in green) and the right temporo-temporal junction. (B) The relationship between Toronto alexithymia scale (TAS) total score and connectivity between the right amygdala seed (in red) and bilateral lingual gyri was moderated by group, with TAS total scores being significantly negatively associated with right amygdala–bilateral lingual gyri connectivity in controls (in blue), but not in fibromyalgia (in yellow). In addition, the right amygdala–bilateral lingual gyri connectivity was significantly weaker in fibromyalgia compared with controls at lower TAS levels (in light green), but stronger at higher TAS levels (in maroon). Compared with controls (in yellow), the fibromyalgia group (in yellow) showed stronger connectivity between the (C) left anterior insula seed and the right posterior insula and (D) between the left anterior cingulate cortex (ACC) seed (in light pink) and the bilateral precuneus and the left inferior parietal lobule. In addition, connectivity between the left anterior insula and the left inferior parietal lobule was significantly and positively associated with TAS total scores. Statistical significance was set at a voxel-wise threshold of *P* = 0.001 uncorrected, to which a family-wise error (FWE) correction was applied on cluster statistics (*p*[*FWEc*] < 0.05). Left is the left of the figure; a.u., arbitrary units; colour-bar represents *t*-statistics; *P < 0.05; **P < 0.01.

#### 3.4.2. Right amygdala seed

The whole-brain multiple linear regression model showed a significant association between the group-by-TAS interaction (*pFWEc* = 0.046) and TAS total scores (*pFWEc* = 0.028), and connectivity between the right amygdala seed and the bilateral lingual gyri during suppression of negative emotions (Table 3B and Fig. 2B). The first moderation analysis (group as moderator) showed that TAS total scores were significantly and negatively associated with right amygdala–bilateral lingual gyri connectivity during suppression on negative emotions in controls (*b* = −0.020, *SE* = 0.006, *t* = −3.145, *P* = 0.003) but not in female participants with fibromyalgia (*b* = 0.005, *SE* = 0.004, *t* = 1.089, *P* = 0.281). The second moderation analysis (TAS total score as moderator) showed that right amygdala–bilateral lingual gyri connectivity was significantly different between the groups at low (*b* = −0.338, *SE* = 0.138, *t* = −2.444, *P* = 0.018; controls > fibromyalgia) and high (*b* = 0.564, *SE* = 0.197, *t* = 2.865, *P* = 0.006; fibromyalgia > controls), but not average levels of TAS total scores (*b* = 0.113, *SE* = 0.096, *t* = 1.171, *P* = 0.247). There was no significant association between connectivity of the right amygdala seed and group.

#### 3.4.3. Left anterior insula seed

The whole-brain multiple linear regression model showed a significant association between group and connectivity between the left anterior insula seed and the right posterior insula (*pFWEc* = 0.011) during suppression of negative emotions (Table 3B and Fig. 2C). During suppression of negative emotions, female participants with fibromyalgia showed heightened left anterior insula–right posterior insula connectivity compared with controls, consistent with increased interoceptive processing. In addition, the TAS total scores were significantly and positively associated with connectivity between the left anterior insula and the left inferior parietal lobule across all participants, independently of group (*pFWEc* = 0.034). There was no significant association between connectivity of the left anterior insula seed and the group-by-TAS interaction.

#### 3.4.4. Right anterior insula seed

There was no significant association between group, TAS total scores or their interaction, and functional connectivity with the right anterior insula seed (Table 3B).

#### 3.4.5. Left anterior cingulate cortex seed

The whole-brain multiple linear regression model showed a significant association between group and connectivity between the left ACC seed and bilateral precuneus (*pFWEc* = 0.012) and the left inferior parietal lobule (*pFWEc* = 0.012) during suppression of negative emotions (Table 3B and Fig. 2D). Female participants with fibromyalgia showed heightened left ACC–bilateral precuneus and left ACC–left inferior parietal lobule connectivity compared with controls. Connectivity of the left ACC seed was not significantly associated with TAS total scores and the group-by-TAS interaction.

#### 3.4.6. Right anterior cingulate cortex seed

There was no significant association between group, TAS total scores or their interaction, and functional connectivity with the right ACC seed (Table 3B).

## 4. Discussion

This study examined the neural correlates of negative emotion suppression in women with fibromyalgia and the moderating role of alexithymia on brain activation and functional connectivity. Compared with controls, the fibromyalgia group showed weaker activation in the left middle frontal gyrus, reduced left amygdala–right TPJ connectivity, and heightened connectivity between the left anterior insula and right posterior insula, and between the left ACC and both the bilateral precuneus and left inferior parietal lobule. Group-by-alexithymia interactions were associated with right amygdala–bilateral lingual gyri connectivity, with opposite patterns in controls and fibromyalgia. In addition, alexithymia was positively associated with left anterior insula–left inferior parietal lobule connectivity across participants. These findings suggest that altered prefrontal, limbic, and interoceptive network activity in fibromyalgia, together with the moderating effects of alexithymia, may reflect disruptions in neural processes underlying emotion identification, integration, and regulation. In this context, reduced prefrontal activation and altered connectivity patterns are interpreted as reflecting less efficient top-down regulation, whereas increased connectivity within interoceptive and self-referential networks is interpreted as maladaptive or compensatory engagement.

From a mechanistic perspective, emotion suppression relies on recognising affective states, engaging prefrontal control regions, and modulating limbic and interoceptive responses. Our findings align with the understanding that alexithymia, characterised by reduced emotional awareness and impaired integration of bodily signals into conscious emotional representations,^12^ may compromise these processes. Neurocognitive models conceptualise alexithymia as a disruption in interoceptive-affective mapping within salience and prefrontal networks,^12,73^ leading to inefficient regulation strategies such as suppression. In this framework, altered functional connectivity during suppression may reflect compensatory or maladaptive engagement of interoceptive and self-referential systems rather than engaging the prefrontal systems that normally provide effective top-down control.

Compared with controls, fibromyalgia participants exhibited reduced activation in the left middle frontal gyrus, a core part of the dlPFC, important for cognitive control and emotion regulation.^17^ Reduced dlPFC recruitment during suppression may reflect impaired top-down modulation of limbic responses, contributing to affective reactivity and limited behavioural flexibility in emotionally salient contexts.^53^ Strengthening dlPFC engagement through emotion regulation–focused psychological approaches^51,52^ or neuromodulation such as repetitive transcranial magnetic stimulation (rTMS)^86^ may normalise dlPFC-related function and enhance adaptive emotional and behavioural responses in fibromyalgia.

Beyond regional activation, group differences emerged in task-related functional connectivity. Women with fibromyalgia exhibited reduced connectivity between the left amygdala and the right TPJ. While no fibromyalgia study has directly examined amygdala–TPJ coupling, reduced connectivity here may reflect a breakdown in integration of affective and higher-order social-cognitive processes. The TPJ supports functions such as self–other differentiation and mentalising^2^ and interacts with the amygdala to enable flexible interpretation and modulation of emotional experiences.^49,54^ Impaired connectivity may limit contextualisation of negative stimuli during suppression, potentially contributing to emotion regulation difficulties in fibromyalgia.^16^ Participants with fibromyalgia also showed increased connectivity between the left anterior insula and right posterior insula, and between the left ACC and the precuneus and left inferior parietal lobule while suppressing negative emotions. These patterns are consistent with heightened interoceptive and self-referential monitoring, which has been linked to bodily hypervigilance in fibromyalgia.^69^ The posterior insula encodes bodily signals, while the anterior insula integrates these into conscious awareness.^20^ Hyperconnectivity between the anterior and posterior insula may amplify somatic sensations and their emotional salience.^25,48^ Similarly, increased ACC–precuneus coupling, core node of the default mode network, may reflect excessive self-referential processing and reduced disengagement from internal states during suppression.^36^ Together, these findings align with models such as FITSS that emphasise maladaptive integration of interoceptive, salience, and self-focused networks.^55^ They highlight specific network interactions, particularly between the salience and default-mode networks, that may underlie hypervigilance and regulatory inefficiency in fibromyalgia. Such patterns suggest candidate neural mechanisms that may inform future intervention studies targeting emotion regulation in fibromyalgia.

Alexithymic traits exerted a differential impact on functional connectivity among the groups. In controls, higher alexithymia scores correlated with weaker right amygdala–bilateral lingual gyri connectivity, regions implicated in visual imagery and emotional encoding,^54^ potentially reflecting reduced visual–emotional integration consistent with prior nonclinical findings.^62^ This association was absent in individuals with fibromyalgia, suggesting that, during suppression of negative emotions, alexithymia disrupts visual imagery components typically supporting emotion suppression. These alterations align with evidence linking alexithymia to heightened ACC recruitment and reduced engagement of interoceptive and self-referential regions such as the insula and precuneus, which has been associated with reduced emotional awareness.^73^ Across all participants, alexithymia was positively associated with stronger left anterior insula–left inferior parietal lobule connectivity, suggesting heightened self-focused processing consistent with maladaptive cognitive styles and prior evidence of altered connectivity between regulation regions and the inferior parietal lobule.^47^ These patterns may help inform future research on neural and behavioural mechanisms supporting emotional awareness and regulation in alexithymia, particularly in fibromyalgia.

Together, the results highlight potential targets for intervention by revealing disrupted integration across emotional, interoceptive, and self-referential networks in fibromyalgia. Interventions enhancing interoceptive accuracy include mindfulness-based approaches.^4,34,77^ Emotional clarity can be improved through emotion labelling or affective education, effective in higher emotional awareness. Cognitive regulation can be strengthened via reappraisal training or acceptance-based strategies.^21,34^ Interventions targeting dlPFC engagement, including emotion regulation–focused psychological approaches,^51,52^ or neuromodulation such as rTMS over the left dlPFC,^86^ may be relevant to future studies examining modulation of prefrontal control in fibromyalgia. In addition to supporting the dlPFC as a stimulation target, the present findings highlight novel network nodes relevant to emotion suppression in fibromyalgia. Reduced left amygdala–right TPJ connectivity and alexithymia-dependent alterations in amygdala–lingual gyrus coupling point to posterior associative and visual regions as potential targets for network-based neuromodulation. Moreover, heightened anterior insula–posterior insula and ACC–precuneus connectivity suggest maladaptive interoceptive and self-referential engagement, raising the possibility that inhibitory modulation of insular or medial frontal hubs may be beneficial. Home-based anodal tDCS over the left dlPFC can modulate ACC–insula connectivity, suggesting a potential avenue for normalisation of maladaptive dynamics.^41^ Future studies may examine whether such interventions influence bodily hypervigilance, emotional awareness, and emotion regulation in fibromyalgia, particularly among individuals with high alexithymia.

Several limitations should be noted. First, the study focused exclusively on suppression, limiting conclusions about broader emotion regulation difficulties in fibromyalgia. Other strategies, such as reappraisal, avoidance, or acceptance, may engage distinct neural systems,^46,65^ consistent with the maladaptive (avoidance, suppression) or adaptive (appraisal) nature of these strategies.^37^ Second, reliance on TAS total score incompletely captures alexithymia's multidimensional structure. Newer tools like the Perth Alexithymia Questionnaire may offer more precise assessment in chronic pain.^1,58^ Third, focusing solely on negative suppression overlooks regulation of positive affect and ambivalent states, also relevant in pain.^10,46^ Fourth, the sample comprised only women, restricting generalisability. Fifth, the high prevalence of post-traumatic stress disorder in fibromyalgia,^76^ and overlapping salience, default mode, and limbic circuits in both conditions,^3,83^ may confound findings and warrant consideration in future research. Finally, the relatively small sample size limits the statistical power of this study, with connectivity results not surviving Bonferroni correction. Brain-wide association work shows reproducible brain–behaviour effects typically require much larger samples or optimised study designs (eg, thousands).^42^ Recruiting such clinical populations remains challenging; only large consortia such as the Enhancing NeuroImaging Genetics through Meta-Analysis (ENIGMA)-Chronic Pain working group^59^ can achieve this. Finally, the cross-sectional design of this study precludes causal inference.

Taken together, the findings indicate that negative emotion suppression in fibromyalgia is characterised by disrupted coordination between emotional, interoceptive, and self-referential networks. Alexithymia further modulated some of these responses, amplifying interoceptive reactivity and limiting emotional clarity. Overall, the results support a model where altered salience and default mode network dynamics contribute to regulation difficulties in fibromyalgia, pointing to potential targets for intervention. Future studies may examine whether interventions such as neuromodulation, mindfulness, emotional awareness training, or cognitive regulation influence these network dynamics and associated emotion regulation difficulties in fibromyalgia, particularly among individuals experiencing alexithymia.

## Conflict of interest statement

The authors have no conflict of interest to declare.

## Supplemental digital content

Supplemental digital content associated with this article can be found online at https://links.lww.com/PAIN/C570.

## Supplementary Material
