# Supplementary material for: Alexithymia moderates brain function during suppression of negative emotions in fibromyalgia
Source: Pain. 2026 Sep 1;167(11):2582–92. doi: 10.1097/j.pain.0000000000004109 (PMC13714771; doi:10.1097/j.pain.0000000000004109)
Supplement: Supplementary file 1 [file jop-167-2582-s001.pdf]

Alexithymia moderates brain function during suppression of negative emotions in fibromyalgia

– Supplementary Material –

Amaia Aguirre de Cárcer Vidal[1,2,3], Nell Norman-Nott[1,2], Sylvia M. Gustin[1,2], Yann Quidé[1,2,*]

[1] NeuroRecovery Research Hub, The University of New South Wales (UNSW) Sydney, Sydney, NSW, Australia

[2] Centre for Pain IMPACT, Neuroscience Research Australia, Randwick, NSW, Australia

[3] School of Psychology and Counselling, Queensland University of Technology, Brisbane, QLD, Australia

*Corresponding author:

Dr Yann Quidé, NeuroRecovery Research Hub, School of Psychology, Biological Sciences (Biolink) building, Level 1, UNSW Sydney, NSW, 2052, Australia.

Tel: +61 2 9065 1883

Email: y.quide@unsw.edu.au ; yannquide@gmail.com

Supplementary Results

Behavioural results

At the end of each block, participants were asked to describe the intensity and valence of their current subjective activation/arousal (How awake do you feel at this moment?”), emotional state (“How do you feel at this moment?”), and physical pain at that moment (“How intense is the pain at this moment?”). During reappraise conditions, participants were instructed to reinterpret the emotional content of the images to reduce their emotional impact. The ‘emotional state’ VAS reflects the subjective intensity of emotional experience (from low to high), not emotional valence.

Table S1. Behavioural results

	Controls (n=30)		Fibromyalgia (N=27)		Statistics		
	Mean	SD	Mean	SD	Welch	df	*p*-value
**VAS Subjective Activation/Arousal**							
Attend neutral	5.424	0.983	4.938	1.361	1.648	56.343	0.105
Attend positive	5.596	1.260	5.583	1.830	0.032	54.844	0.974
Attend negative	**5.586**	**1.225**	**6.458**	**1.777**	**-2.298**	**54.854**	**0.025**
Reappraise positive	5.657	1.324	5.969	1.658	-0.837	59.253	0.406
Reappraise negative	5.667	1.164	5.844	1.698	-0.489	54.689	0.627
Suppress positive	5.475	1.261	5.615	1.245	-0.450	62.979	0.654
Suppress negative	5.667	1.310	5.979	1.788	-0.802	56.765	0.426
**VAS Emotional State**							
Attend neutral	5.697	1.245	5.313	1.230	1.252	62.978	0.215
Attend positive	7.071	1.592	6.375	1.656	1.726	62.683	0.089
Attend negative	**3.818**	**1.852**	**2.750**	**1.737**	**2.399**	**62.932**	**0.019**
Reappraise positive	7.657	1.464	7.219	1.556	1.168	62.470	0.247
Reappraise negative	**4.535**	**1.648**	**3.490**	**1.604**	**2.593**	**62.999**	**0.012**
Suppress positive	6.323	1.494	5.906	1.331	1.189	62.560	0.239
Suppress negative	**4.273**	**1.519**	**3.135**	**1.800**	**2.749**	**60.611**	**0.008**
**VAS Pain**							
Attend neutral	**0.970**	**1.690**	**5.240**	**2.438**	**-8.182**	**55.045**	**<0.001**
Attend positive	**0.778**	**1.363**	**5.073**	**2.628**	**-8.232**	**46.237**	**<0.001**
Attend negative	**0.859**	**1.850**	**6.313**	**2.444**	**-10.122**	**57.759**	**<0.001**
Reappraise positive	**0.838**	**1.720**	**5.021**	**2.685**	**-7.452**	**52.523**	**<0.001**
Reappraise negative	**0.960**	**2.061**	**5.896**	**2.410**	**-8.862**	**60.891**	**<0.001**
Suppress positive	**0.596**	**1.416**	**5.385**	**2.471**	**-9.547**	**49.062**	**<0.001**
Suppress negative	**0.980**	**1.639**	**5.813**	**2.445**	**-9.331**	**53.979**	**<0.001**

VAS: visual analogue scale score; SD: standard deviation; df: degrees of freedom

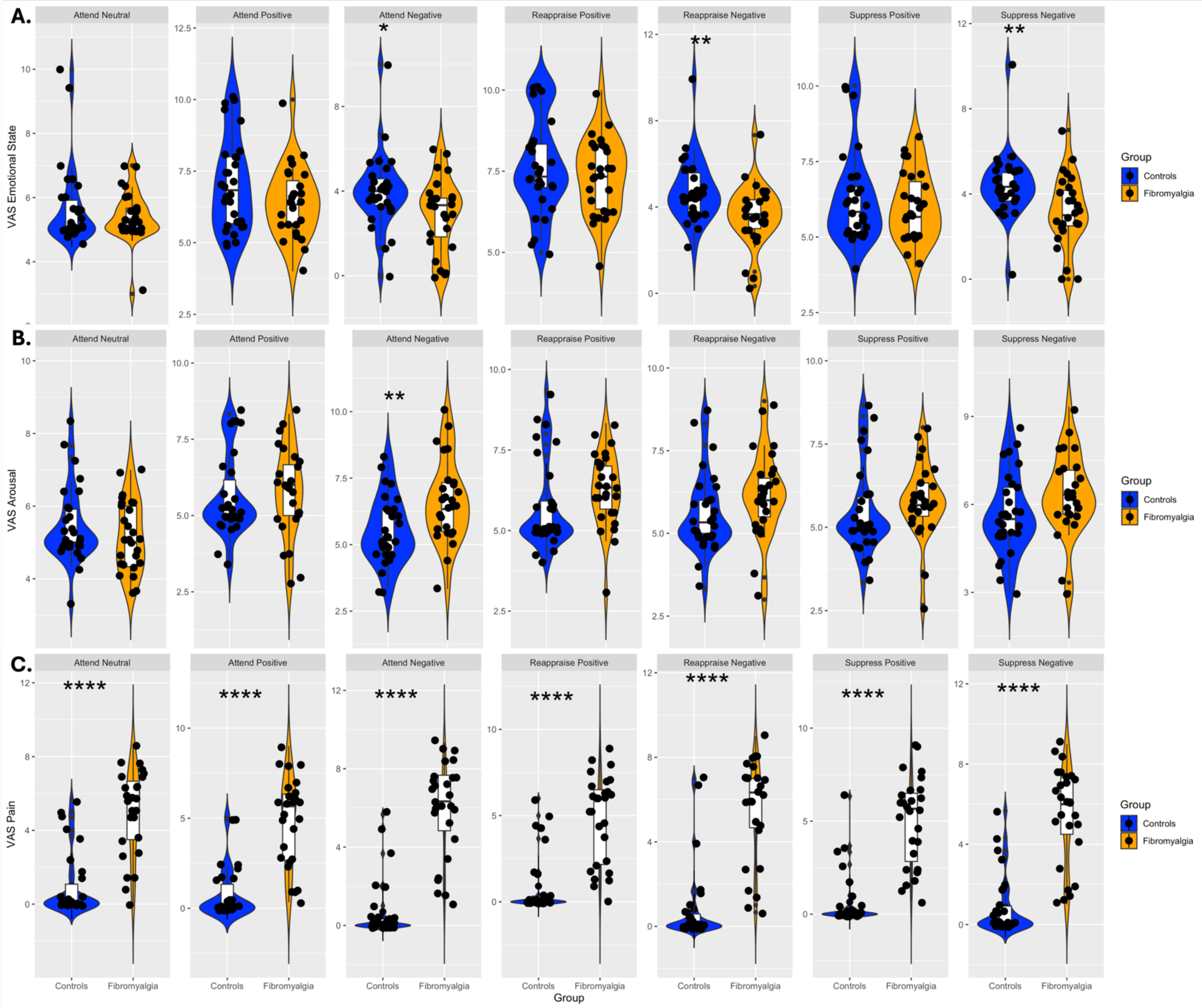

Figure S1. Representation of the behavioural results

Group differences between the fibromyalgia (orange on the right side) and control groups (in blue, on the left) on the visual analogue scales (VAS) for (A) emotional state, (B) subjective activation/arousal, and (C) pain intensity for all task conditions, from left to right: attend neutral, attend positive, attend negative, reappraise positive, reappraise negative, suppress positive and suppress negative emotions. Reappraise conditions required participants to reinterpret images to reduce emotional impact; the 'emotional state' VAS indexes subjective emotional intensity rather than emotional

valence. The fibromyalgia group reported significantly lower levels of emotional state (that is, feeling less emotionally well) during conditions involving negative emotions (attend, reappraise, suppress) compared to controls, and reported higher levels of subjective activation/arousal (that is feeling more awake) while attending negative emotions. The fibromyalgia group reported significantly higher levels of pain during all conditions compared to controls.

*$p < 0.05$, **$p < 0.01$, ****$p < 0.0001$
